# What training should psychiatrists have to interpret six- and 12-lead electrocardiograms?

**DOI:** 10.1192/bjb.2022.87

**Published:** 2023-12

**Authors:** George Crowther, Mani S. Krishnan, Jonathan Richardson, Robert Bowes, Andrew Fitzpatrick, Muzahir H. Tayebjee

**Affiliations:** 1 Leeds and York Partnership NHS Foundation Trust, UK; 2 University of Leeds, UK; 3 Tees, Esk and Wear NHS Foundation Trust, UK; 4 Tyne and Wear NHS Foundation Trust, UK; 5 Leeds General Infirmary, UK

**Keywords:** Antipsychotics, electrocardiogram, QTc, interpretation, six-lead ECG

## Abstract

To monitor for drug-related cardiac arrhythmias, psychiatrists regularly perform and interpret 12-lead (12L) and, increasingly often, six-lead (6L) electrocardiograms (ECGs). It is not known how training on this complex skill is updated or how well psychiatrists can interpret relevant arrhythmias on either device.

We conducted an online survey and ECG interpretation test of cardiac rhythms relevant to psychiatrists.

A total of 183 prescribers took part; 75% did not regularly update their ECG interpretation skills, and only 22% felt confident in interpreting ECGs. Most participants were able to recognise normal ECGs. For both 6L and 12L ECGs, the majority of participants were able to recognise abnormal ECGs, but fewer than 50% were able to correctly identify relevant arrhythmias (complete heart block and long QTc). A small number prescribed in the presence of potentially fatal arrhythmias. These findings suggest a need for mandatory ECG interpretation training to improve safe prescribing practice.

People with severe mental illness are at a greater risk of cardiovascular disease.^1^ Furthermore, some drugs prescribed in psychiatry can cause disruption to cardiac electrical activity. Commonly described phenomena include: prolongation of the time for ventricular depolarisation and repolarisation to occur, represented as QT prolongation on an ECG; partial or complete atrioventricular conduction block, causing PR prolongation on the ECG; and delayed ventricular electrical activation, causing QRS prolongation on the ECG. In severe cases, these conduction abnormalities can lead to life-threatening cardiac arrhythmias: QTc prolongation causing torsade de pointes;^2^ atrioventricular conduction delay indicating first-, second- or third-degree heart block;^3^ and prolonged QRS complex associated with sudden cardiac death.^4^

Examples of commonly prescribed psychotropic medications and their associated cardiac conduction abnormality are listed below.

- Antipsychotics, some antidepressants (citalopram, escitalopram and tricyclics) and methadone – QT prolongation.^5^
- Antipsychotic-associated ventricular arrhythmia and sudden cardiac death following QTc prolongation (1.53-fold relative risk).^6^
- Acetylcholinesterase inhibitors (AChEIs) – atrioventricular block.^7^
- Tricyclic antidepressants – QRS prolongation.^5^

Baseline and follow-up 12-lead (12L) ECG can screen for patients who are vulnerable to cardiac arrhythmias, and this is recommended before prescribing an antipsychotic when patients have a history of cardiovascular disease or when high doses are required.^8^ Most mental health trusts in the UK also aim to complete a ‘baseline’ ECG on every patient admitted to an in-patient ward.^9^ This is in case they need urgent antipsychotics at a time when ECG is impractical (owing to agitation, for example). In most cases, the ECGs will be interpreted by a psychiatrist, either a consultant or a more junior-grade doctor.

Guidance on when to perform ECGs before commencing AChEIs is less well described. Nonetheless, they are commonly performed and interpreted by psychiatrists.^10,11^

The upshot of the guidelines and practice described above is that ECG interpretation is a common part of many psychiatrists working week. ECG interpretation is a skill that requires ongoing continuous professional development for all doctors,^12^ yet it is rarely mandated for psychiatrists,^11^ despite evidence to suggest it is an area of specialty weakness.^13^

Novel handheld ECG monitors are becoming increasingly popular. They can produce a six-lead (limb leads) ECG (6L) equivalent and are readily available to healthcare professionals and the public. These 6L devices are practically advantageous over 12L; they are small (credit-card sized), portable and do not require exposing the patient.^14^ The patient places their fingers or thumbs on the device's top sensors while resting it on exposed skin of the knee or ankle, allowing a sensor on the bottom to make electrical contact. The recorder is connected via Bluetooth to a tablet or smart phone, where the ECG can be reviewed. They do not yet provide automated readouts for PR interval, QRS duration, QT or QTc duration. However, they have recently been validated for this,^14^ and one device (Kardia 6L) has received US Food and Drug Administration approval for QT assessment. These devices are increasingly ubiquitous and are already used by some psychiatrists. They are also the topic of a NICE guideline that is in development; evaluating the Kardia 6L recorder for measuring QT interval in people receiving antipsychotic medication.

Given that 12L interpretation is such a core part of psychiatric prescribing practice, and with the possible emergence of 6L ECG machines, we devised a study to investigate the training, confidence and skills of psychiatrists to interpret ECGs relevant to psychiatry prescribing using both 6L and 12L machines.

The aims of the study were as follows:

- to describe psychiatrists’ confidence at interpreting ECGs relevant to psychiatry;
- to describe how regularly psychiatrists update ECG interpretation training;
- to describe and compare the accuracy with which psychiatrists can interpret ECGs relevant to prescribing in psychiatry.

## Method

Prescribers in psychiatry who regularly interpret ECGs (including nurse and pharmacy prescribers) from a range of subspecialties, in differing job roles and from different areas of the country were invited to participate in a free online ECG interpretation test and learning event. To ensure participants were from a wide range of subspecialties and professional backgrounds, with varying levels of confidence in ECG interpretation, a sampling frame was used.

The event was hosted by a national provider of psychiatry learning and development. It was advertised via email to their subscribers. In the promotional material, participants were informed that they would be asked to take part in an ECG interpretation test.

To provide an opportunity for more people to attend, two identical events were held, 1 month apart. Each event was live and lasted 1 h. All participants were given a short introduction to the session, and it was reiterated that there would be an interpretation test and that the results would be analysed and written up for publication. Participation in the test was voluntary, participants could abstain from all or part of the test and still attend the subsequent teaching, and participation subsequently was taken as implied consent. The test then commenced (prior to any teaching on the topic).

Online polling software ‘Vevox’ was used to capture responses.^15^ All responses were anonymous, and all questions are published in the Supplementary material accompanying this article. Participants were first asked to rate on a Likert scale how confident they were at interpreting ECGs. They were then asked about their experience using 6L machines and how often, if ever, they updated their ECG interpretation skills (6L or 12L).

Participants were shown ten ECGs in sequence and asked identical questions about each. They had 1 min to analyse the ECG and answer the questions. Participants were shown five common rhythms: normal sinus rhythm (good quality trace), normal sinus rhythm (poor quality trace), atrial fibrillation, QT prolongation and complete heart block. For each rhythm, they were shown a 12L trace and an 6L trace. The 12L and 6L traces were paired but shown in a random order (ECGs are displayed in Supplementary material).

For each ECG, participants were asked to assess the ECG quality and whether the trace was normal or not, select the correct diagnosis from a drop-down list, and describe if in a non-urgent situation they would prescribe an antipsychotic or AChEI in the context of the ECG (if the respondent did not prescribe AChEIs regularly, they were told to skip the last question). To ensure ECGs were relevant, of a sufficient complexity and interpretable in 1 min, they were piloted on a small group of psychiatrists who did not attend the event (*n* = 7). The definition of quality was based principally on the presence or absence of artefacts produced on the trace as a result of poor technique.^16^

Following the test, a 40 min lecture on ECG interpretation for psychiatry prescribers was given by a consultant cardiac electrophysiologist.

The study protocol was reviewed by the Leeds and York Partnership NHS Foundation Trust's clinical effectiveness team, and using the health research authority's online decision tool,^17^ and was approved as national service evaluation.

## Results

### Demographics

A total of 208 people from 27 mental health trusts in England attended the event, and 183 took part in the online test. Twenty-five people abstained from the test and only attended the subsequent ECG lecture. Participants had the option to skip questions, so not all participants answered every question.

Participants were of a range of training grades and subspecialties satisfying the sampling frame:

- consultants, 46% (*n* = 84);
- speciality doctors and associate specialists, 18% (*n* = 33);
- core trainees, 13% (*n* = 25);
- higher trainees, 10% (*n* = 19);
- non-medical prescribers, 8% (*n* = 15);
- foundation-year doctors, 4% (*n* = 7);
- working age, 54% (*n* = 98);
- old age, 25% (*n* = 45);
- liaison, 8% (*n* = 15);
- child and adolescent mental health services, 6% (*n* = 11);
- forensic, 4% (*n* = 8);
- learning disabilities. 3% (*n* = 6).

### ECG interpretation confidence and training updates

Based on 163 responses, no respondent felt very confident interpreting ECG traces, 22% (*n* = 36) felt fairly confident, 30% (*n* = 48) felt neither unconfident nor confident, 48% felt unconfident (*n* = 64) or very underconfident (*n* = 14); 72% (*n* = 118) of respondents had heard of 6L ECG recorders prior to the talk and 28% (*n* = 33) of them had used one. Of those that had used the 6L recorder, 78% (*n* = 26) found them difficult to interpret. Seventy-five per cent (*n* = 121) of respondents did not update their ECG training regularly. Of those that did, 60% (*n* = 24) had done so in the past year.

### ECG interpretation survey

ECGs were shown to participants in a random order; however, they are presented as paired (Table 1 and Supplementary file 1 available at https://doi.org/10.1192/bjb.2022.87).

**Table 1:** Participant ECG interpretation accuracy

	Normal ECG, good quality		Atrial fibrillation, good quality		Complete heart block, good quality		Prolonged QT, good quality		Normal ECG, poor quality	
	12L	6L	12L	6L	12L	6L	12L	6L	12L	6L
Stated quality was good, *n*/*r* (%)	123/152 (81)	141/152 (93)	132/152 (87)	57/157 (36)	124/164 (76)	134/160 (84)	150/157 (96)	130/146 (89)	144/156 (92)	113/135 (84)
Correctly identified ECG normal or abnormal, *n*/*r* (%)	92/152 (61)	121/152 (81)	150/153 (98)	148/156 (95)	152/161 (94)	150/156, (96)	138/155 (89)	122/144 (85)	6/155 (4)	34/134 (25)
Interpreted ECG correctly, *n*/*r* (%)	92/152 (61)	121/152 (81)	84/150 (56)	72/149 (46)	31/161 (20)	37/160 (23)	77/155 (50)	65/146 (45)	6/155 (4)	34/134 (25)
Would prescribe antipsychotics, *n*/*r* (%)	94/145 (65)	116/145 (79)	23/151 (15)	19/158 (12)	21/156 (13)	14/158 (9)	24/142 (15)	24/142 (17)	12/156 (8)	50/132 (38)
Would prescribe AChEI, *n*/*r* (%)	55/84 (65)	65/83 (78)	18/84 (21)	13/92 (14)	6/92 (7)	12/89 (13)	15/89 (16)	12/83 (19)	8/90 (9)	3/79 (39)

*r*, number of total responses to the question.

#### Normal sinus rhythm ECGs

In good-quality 6L and 12L traces, 81% and 63% of respondents accurately recognised normal sinus rhythm, respectively. In poor-quality traces, only 11% (12L) and 15% (6L) identified that the traces were normal, with a resulting impact on prescribing decisions: 92% (12L) and 62% (6L) of respondents withheld antipsychotics, and 91% (12L) and 61% (6L) withheld AChIs.

#### Abnormal good-quality ECGs

The majority of respondents were able to identify that the trace was abnormal for both the 6L and 12L devices with similar accuracy. Diagnostic accuracy was low with both 6L and 12L traces: 85% (12L) and 88% (6L) of respondents incorrectly withheld antipsychotics in the presence of atrial fibrillation; 15% (12L) and 17% (6L) prescribed antipsychotics in the presence of QTc >540, and 7% (12L) and 13% (6L) prescribed AChEIs in cases of complete heart block.

## Discussion

### Summary of main findings

Participants’ confidence in ECG interpretation was generally low. Although most were able to identify normal ECG traces, the majority were not able to diagnose abnormal traces. The impact of interpretation on prescribing practice was striking, with antipsychotics and AChEIs being both withheld overcautiously and prescribed in cases of potentially life-threatening arrhythmias.

### Strengths and limitations

This is the first large-scale description of the ECG skills and confidence of UK psychiatry prescribers. However, this study had a number of limitations. The time-pressured and artificial environment of a test setting may have made responders more likely to err on the side of overreporting the ECG. We removed the computer-generated report from the 12L, as the 6L does not have an automatic report; as a result, we may have seen better responses with the 12L. However, we felt that this was of use, as it is important that the automatic report is not taken for granted and that it is reviewed, as it can be incorrect.

It is also of note that all participants were attending a learning event on ECGs. This has the potential to introduce a selection bias, attracting those where were either underconfident or had an interest in ECG interpretation. To help address this, we demonstrated that participants were from a wide range of backgrounds, with differing levels of experience and self-confidence in ECG interpretation.

The majority of participants were consultant-grade psychiatrists, limiting the generalisability of the results. One might also assume that this group are the furthest removed from their basic medical skills training (of which ECG interpretation would be part) and therefore a cohort that lacks skills and confidence. In practice, they are also a group that may ask for ECGs to be interpreted for them by more junior doctors. Nonetheless, they ought to be able to interpret the trace, as help may not be at hand in an urgent situation.

Twenty-five people at the event abstained from the test. They did not provide a reason for this, but one might conclude they were underconfident to take the test or unable to use the online polling software. This has the potential to introduce further bias and skew the results positively.

### Clinical implications of the results

Psychiatrists are required to obtain and interpret ECGs before initiating certain medications. Traditionally, this has been done using 12L traces. Although the procedure is painless, it can cause embarrassment (owing to exposure) and feel frightening to the uninformed. Further, many psychiatry settings are not equipped to perform the test. Novel 6L handheld ECG recorders are rapidly gaining popularity among clinicians and the public.^18,19^ They may have future clinical utility. However, a 6L or 12L device is only beneficial if its results are interpreted accurately.

Despite all being regular prescribers few participants felt confident at ECG reporting. This may be unsurprising, ECG interpretation is a complex skill that requires training and regular re-validation; many doctors struggle with it.^12^ Despite this however, 75% of participants did not update the skill regularly.

Auto ECG interpretation is standard for most 12L ECG recorders. This functions provides an automated readout of QTc and PR interval. Auto interpretation is currently not standard for 6L monitors except for rhythm recognition. Nevertheless, it is important that clinicians can still read ECGs to validate the computer-generated report and, in the case of 6L recorders, interpret the traces. Accuracy of interpretations from both 6L and 12L recordings were similarly poor; almost 25% of the group did not recognise a normal ECG. Moreover, there was a tendency to overcall ECGs as abnormal and deny patients medication, which could have a negative impact on their mental health. Finally, and more worryingly, a small but not insignificant proportion would prescribe these drugs when contraindicated and not prescribe when indicated. At best, poor ECG reporting could delay treatment and also lead to unnecessary referrals to cardiology. At worst, it could potentiate a fatal arrhythmia, with obvious significant implications for patient safety and resulting medico-legal challenges for the clinician. In practice, where the psychiatrist is underconfident in their ECG reporting skills, they could double-check the result with a more confident colleague, or use an ECG interpretation service if available in their trust.^20^ This has the potential to avert harm, but it is not 100% reliable, as it depends on the skills and availability of a colleague or availability of an interpretation service.

In conclusion, this survey reveals huge variance in ECG skills and interpretation among prescribers of psychiatric drugs, with relative agreement between the 12L and 6L recorders. These findings suggest that whatever ECG recorder is used, if prescribers are unable to confidently interpret the traces because of lack of training, the patient may either be denied treatment unnecessarily or end up suffering harm. Therefore we recommend that psychiatrists are required to update their ECG reporting skills as part of revalidation.

## Supporting information

Crowther et al. supplementary material Crowther et al. supplementary material

## Data Availability

The data that support the findings of this study are available on request from the corresponding author (G.C.). The data are not publicly available owing to their containing information that could compromise the privacy of research participants.

## About the authors

**George Crowther** is a consultant old age psychiatrist at Leeds and York Partnership NHS Foundation Trust and visiting senior lecturer at the Leeds Institute of Health Sciences, University of Leeds, UK; **Mani S. Krishnan** is a consultant in old age/liaison psychiatry at Tees, Esk and Wear NHS Foundation Trust, UK; **Jonathan Richardson**, is a consultant old age psychiatrist and Chief Clinical Information Officer at Tyne and Wear NHS Foundation Trust, Cumbria, Northumberland, UK; **Robert Bowes**, is a specialist cardiac physiologist at the Department of Cardiology, Leeds General Infirmary, Leeds, UK; **Andrew Fitzpatrick** is a clinical scientist and Head of Physiology at the Department of Cardiology, Leeds General Infirmary, Leeds, UK; and **Muzahir H. Tayebjee** is a consultant cardiologist and electrophysiologist at the Department of Cardiology, Leeds General Infirmary, Leeds, UK
