# Supplementary material for: What training should psychiatrists have to interpret six- and 12-lead electrocardiograms?
Source: BJPsych Bull. 2023 Dec;47(6):352–6. doi: 10.1192/bjb.2022.87 (PMC10694687; doi:10.1192/bjb.2022.87)
Supplement: Crowther et al. supplementary material [file S2056469422000870sup001.docx]

|  | 12 Lead ECG | 6 Lead Kardia ECG |
| --- | --- | --- |
| Atrial Fibrillation | 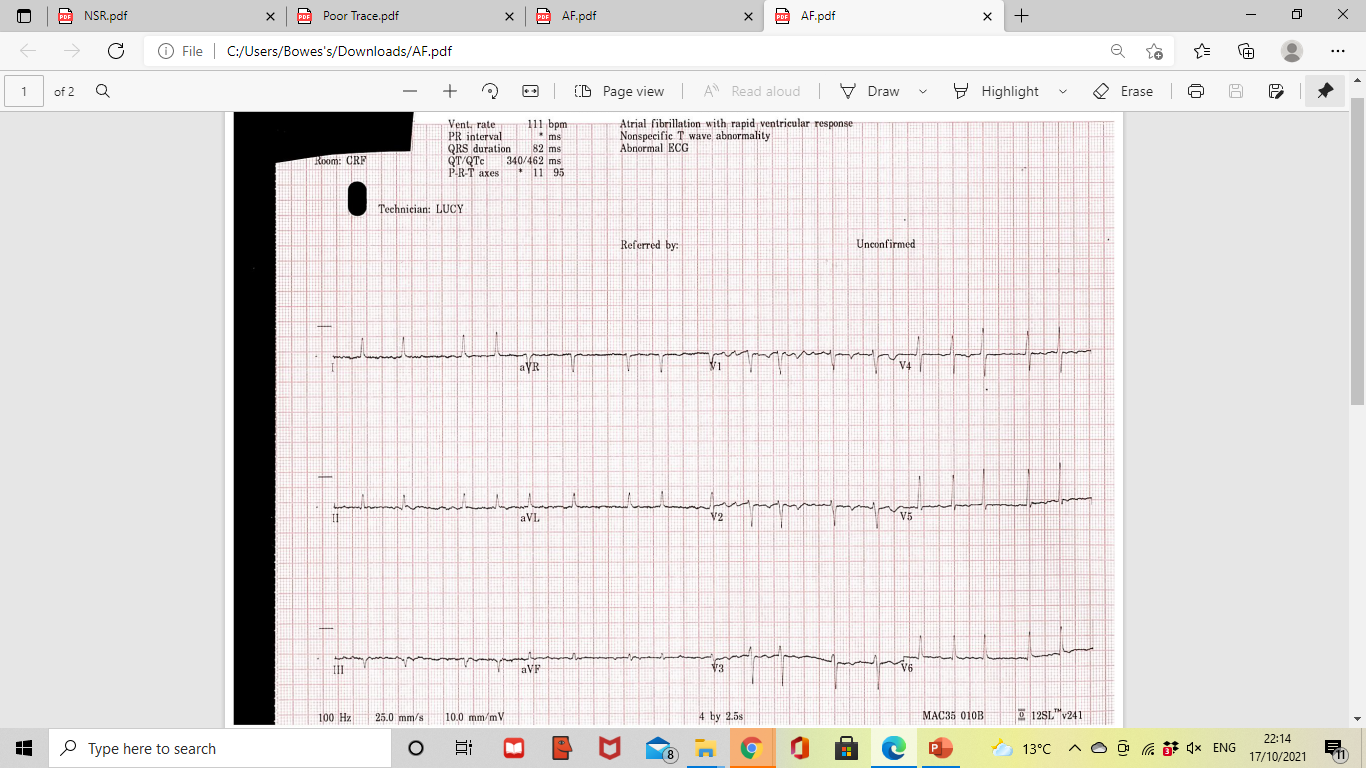 | 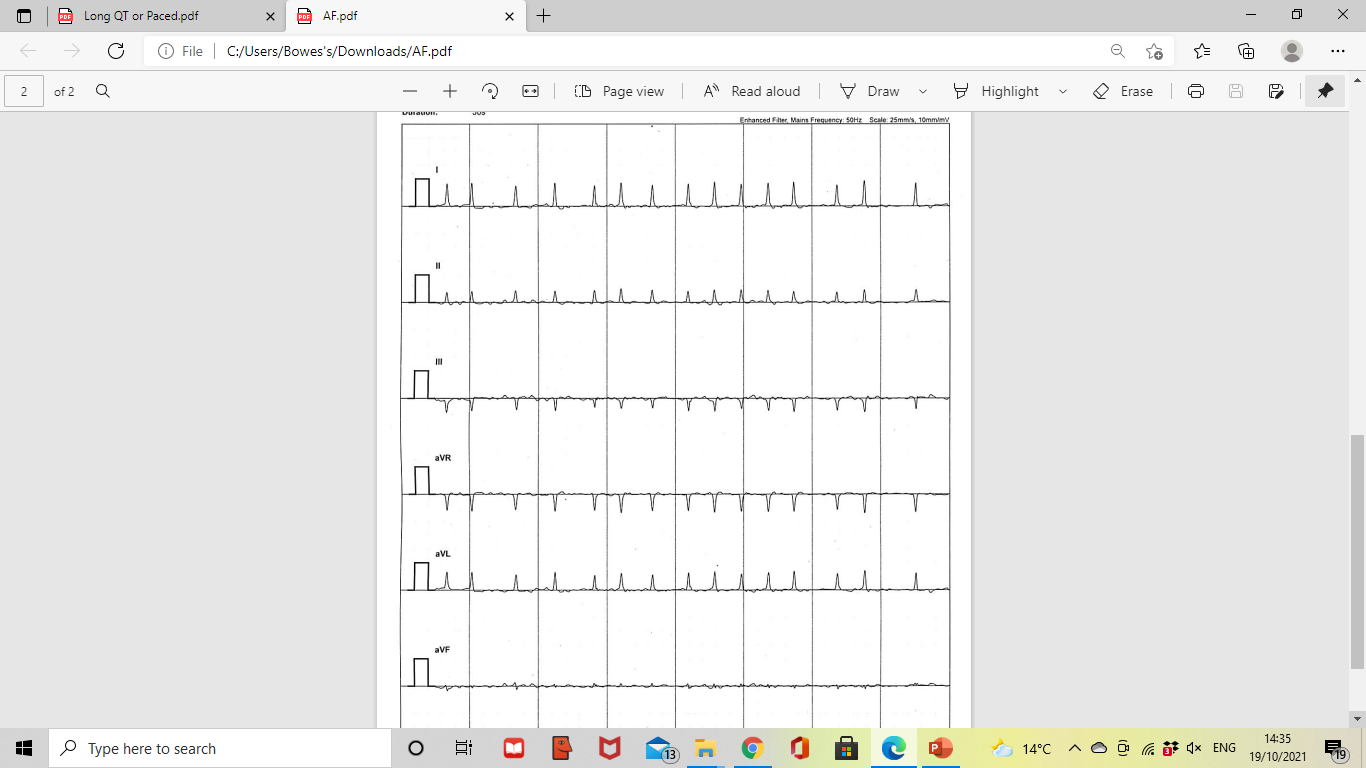 |
| Normal Good Quality ECG | 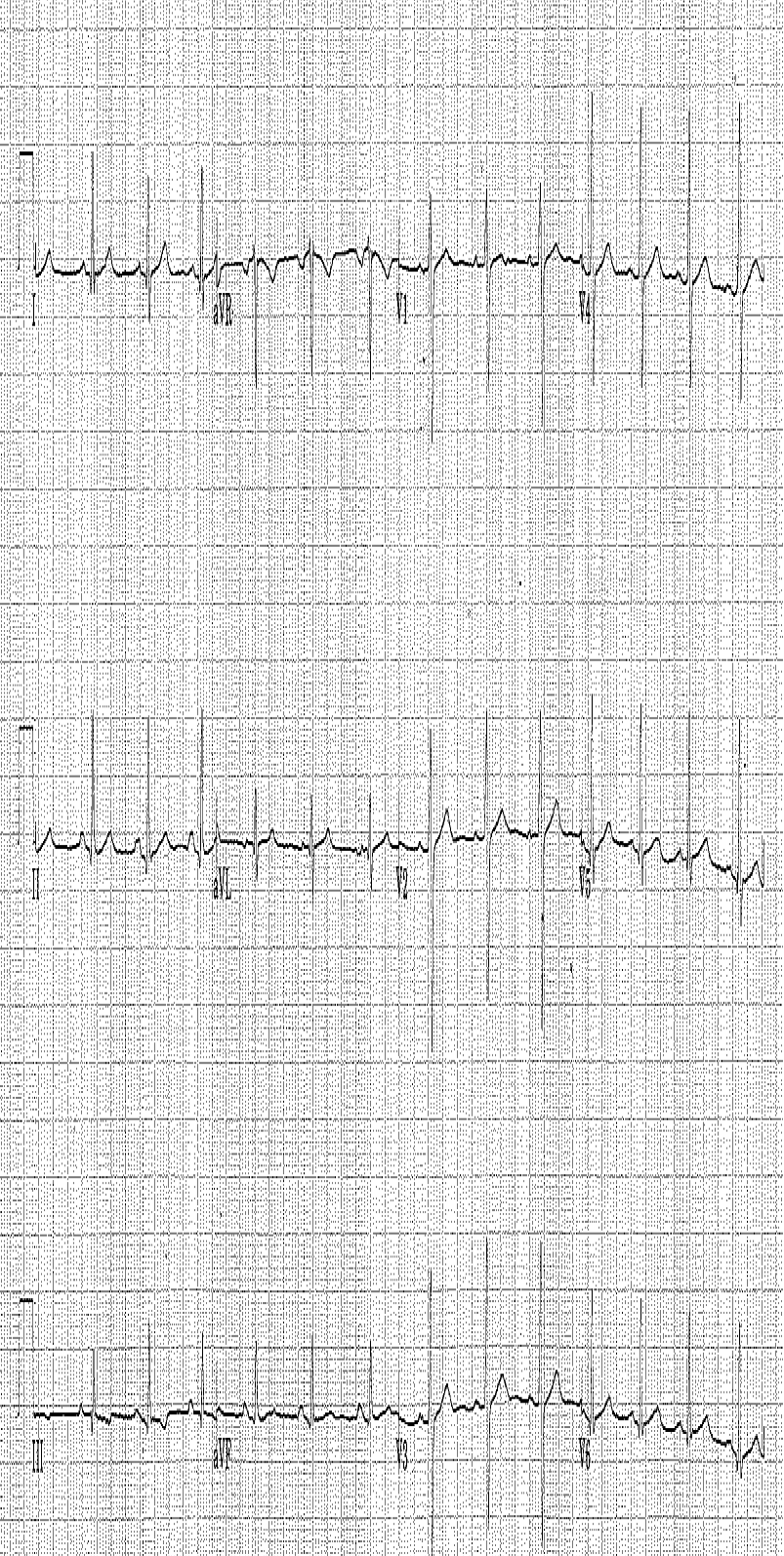 | 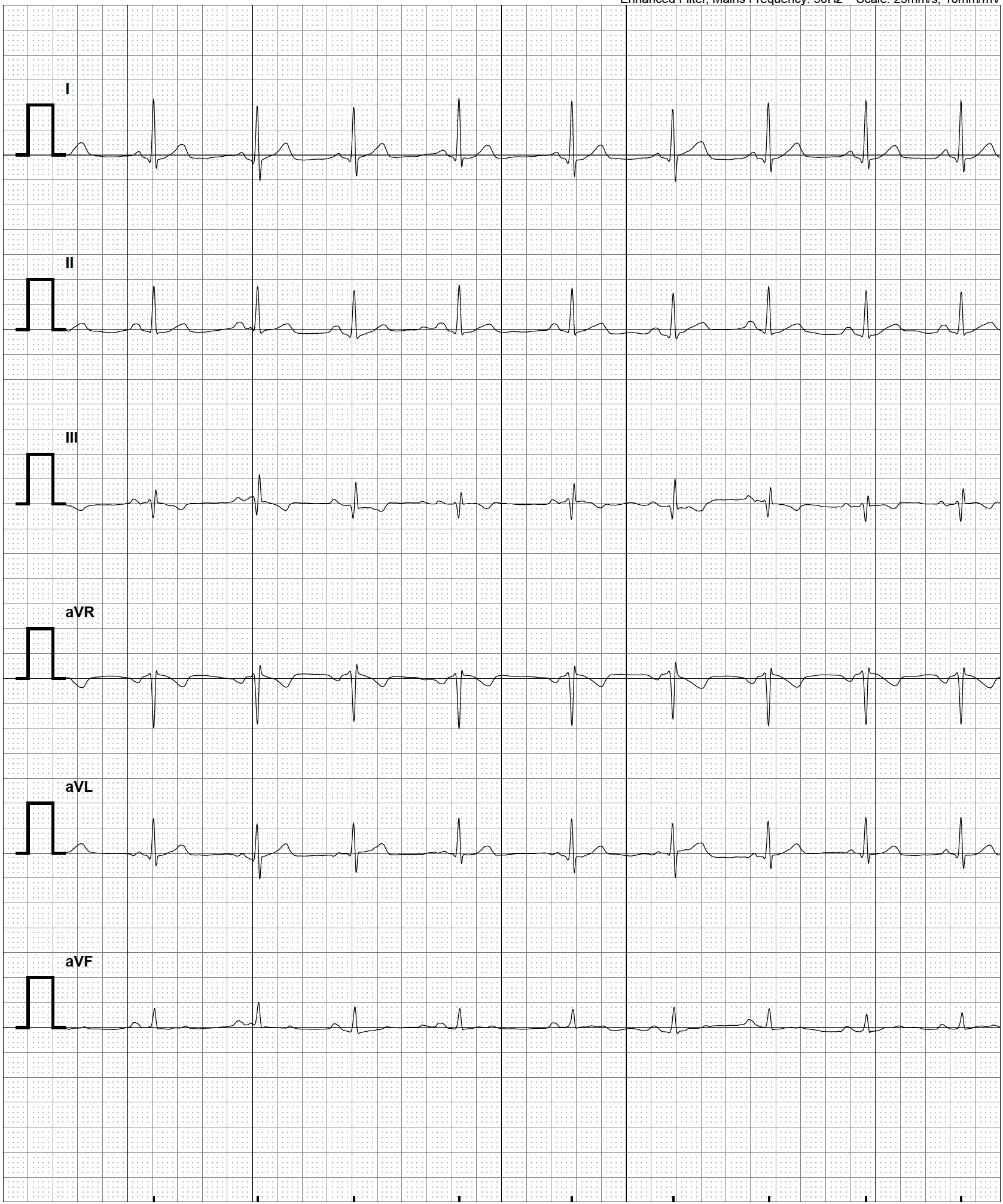 |
| Complete Heart Block | 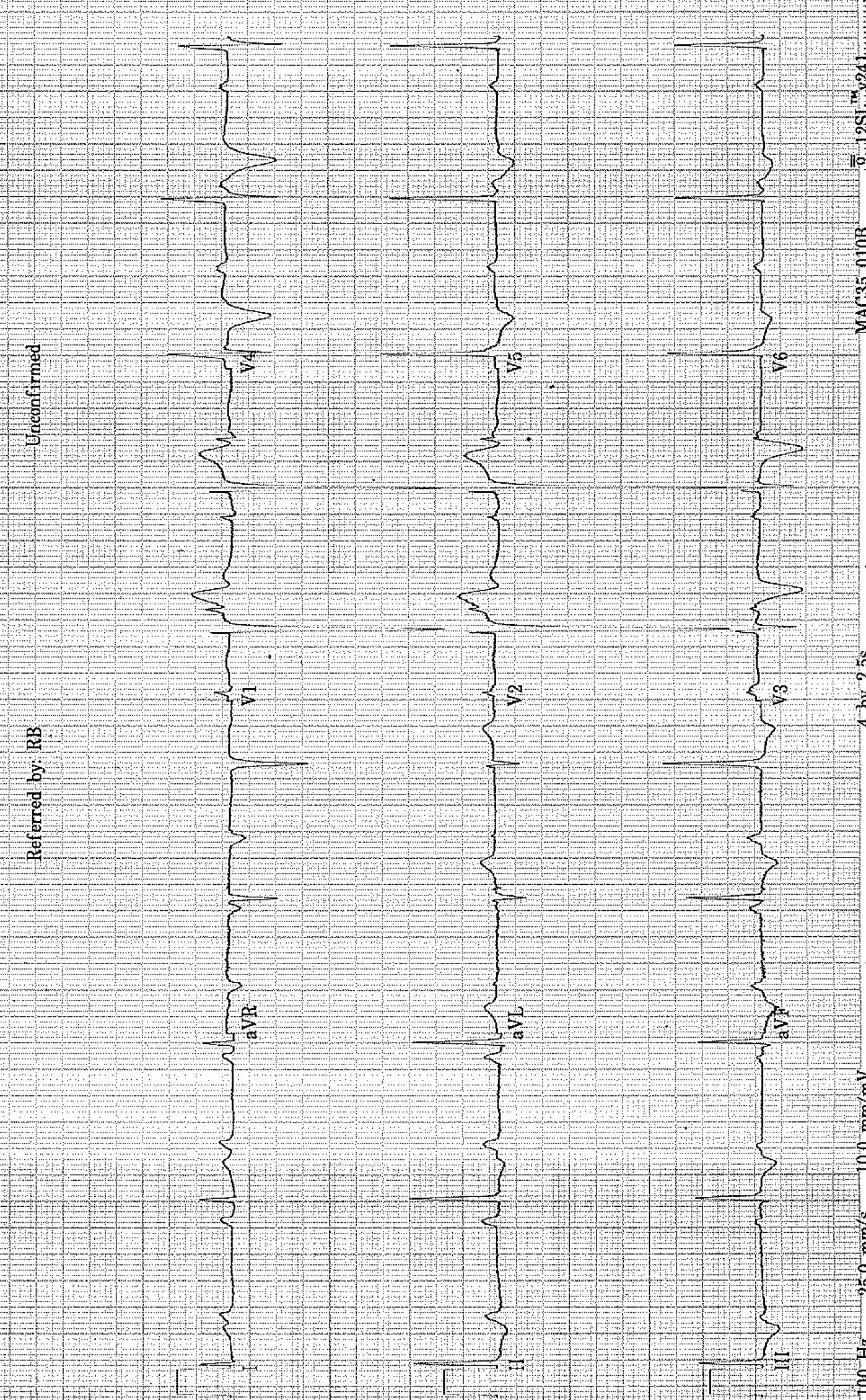 | 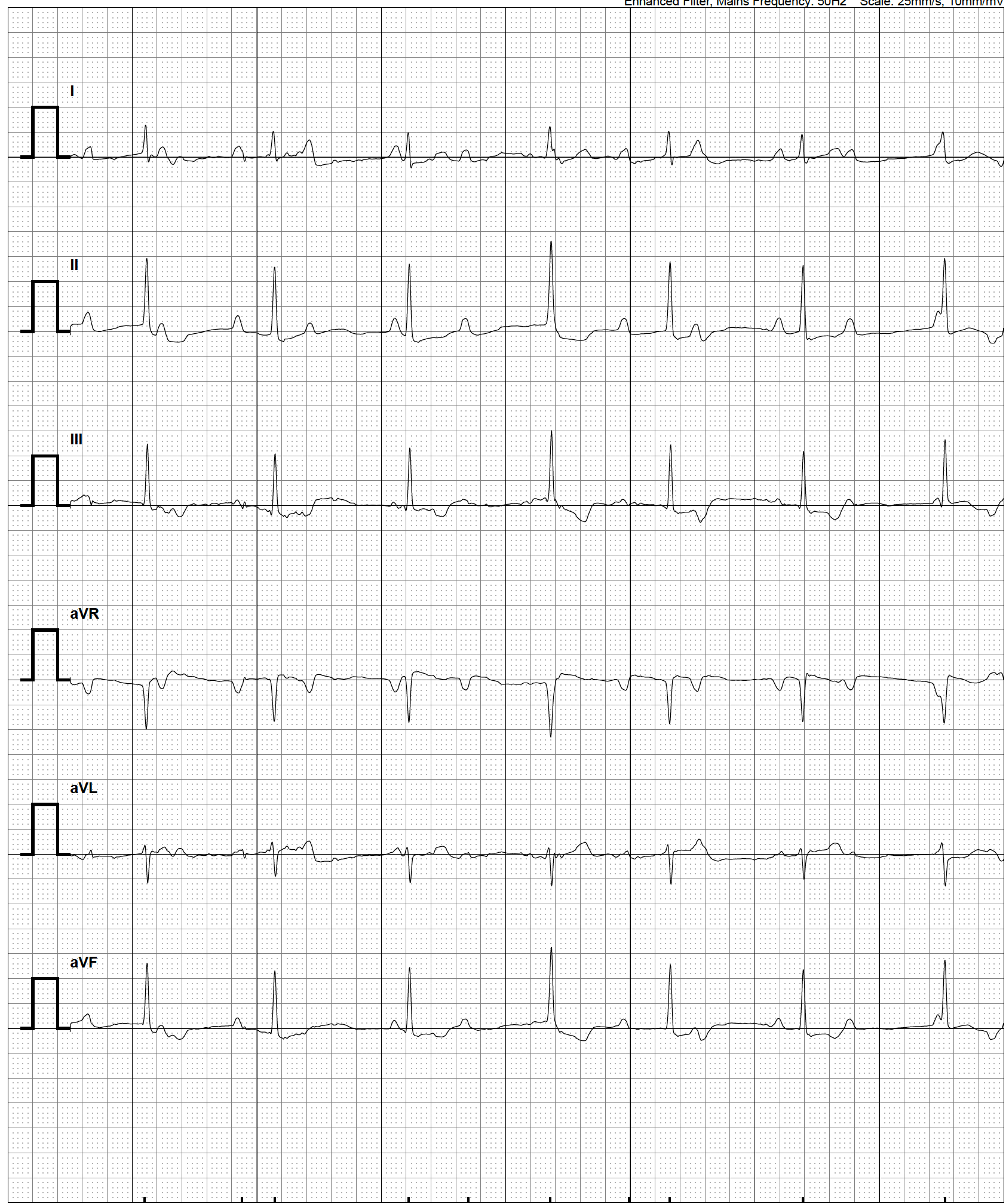 |
| Normal 12 lead with noise and 6L with lead transposition, both classified as poor quality | 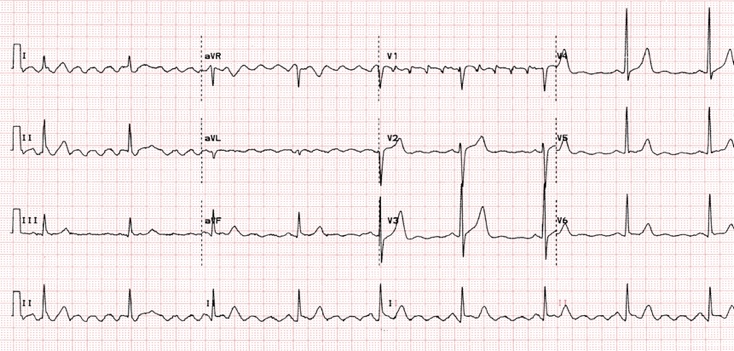 | 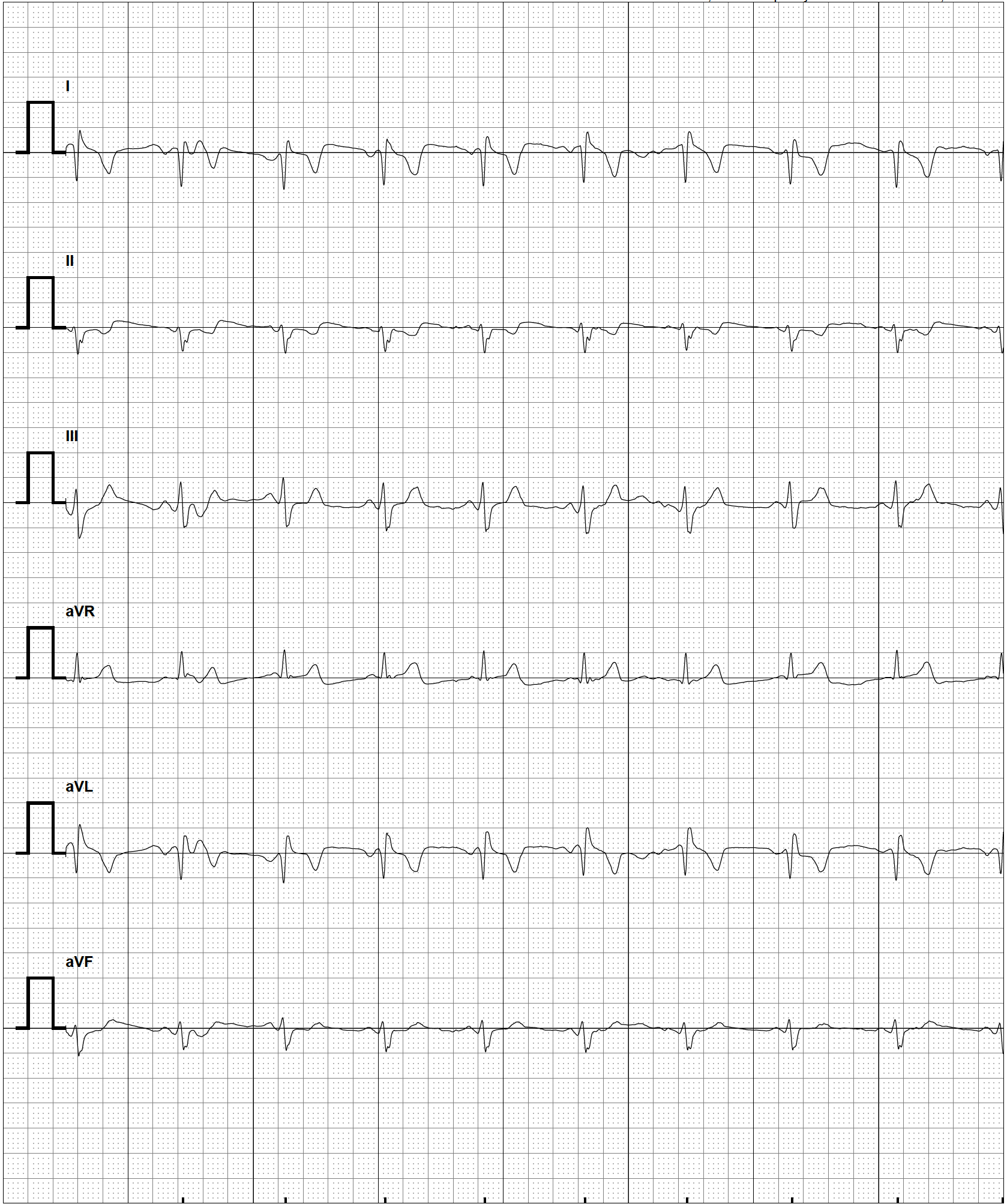 |
| Long QT | 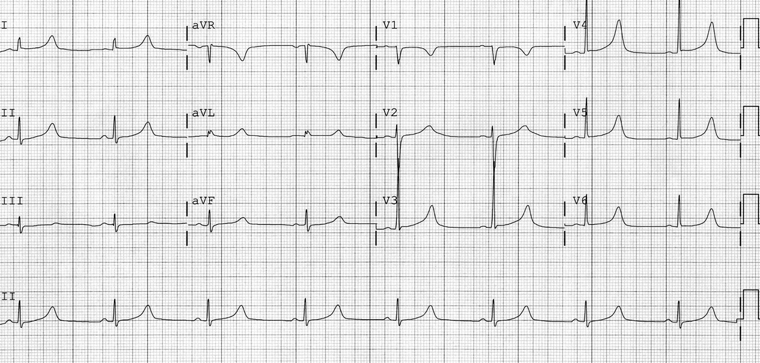 | 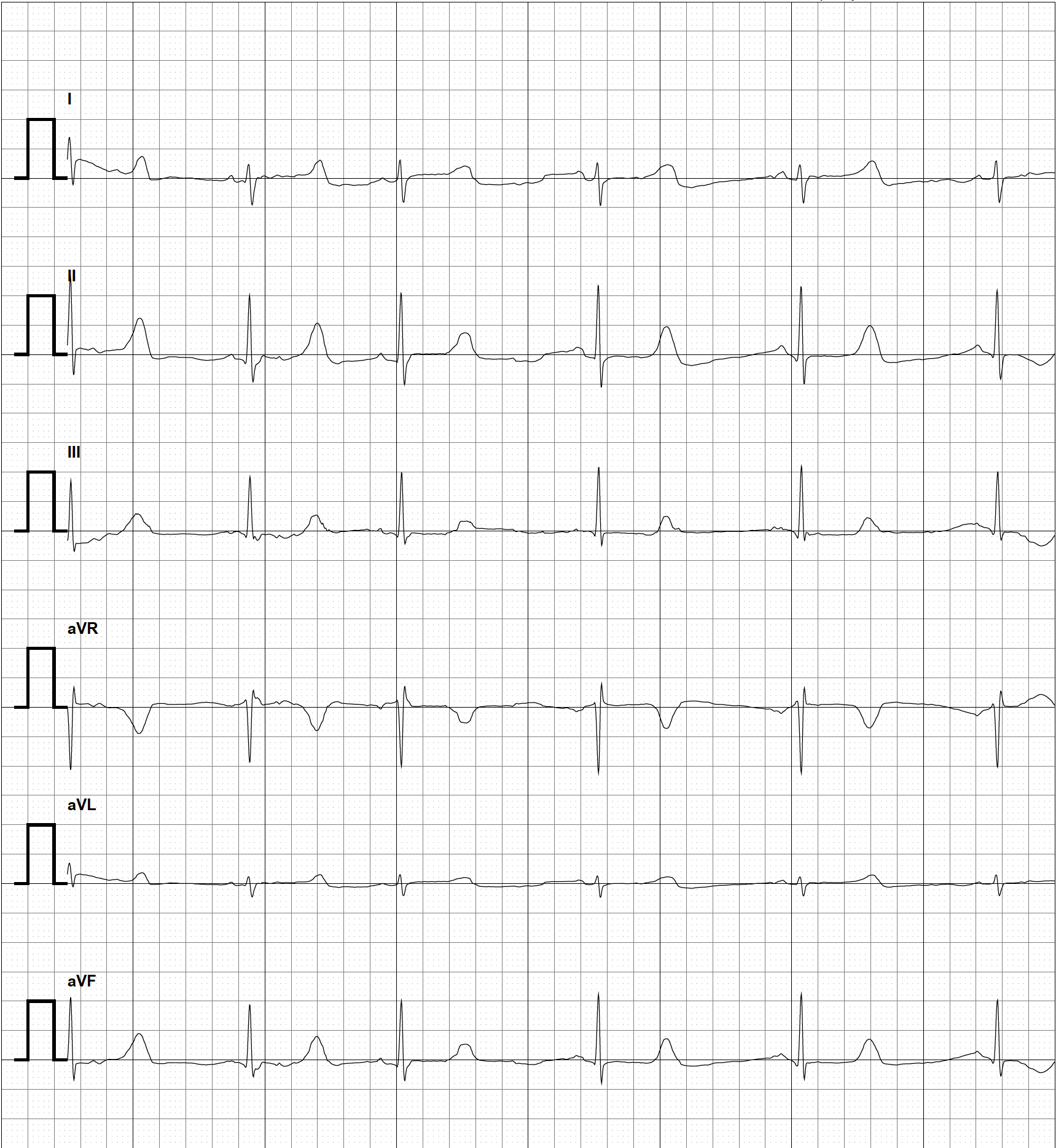 |
